# Interaction of dense breast patterns with other breast cancer risk factors in a case–control study

**DOI:** 10.1038/sj.bjc.6601911

**Published:** 2004-06-08

**Authors:** S W Duffy, R W Jakes, F C Ng, F Gao

**Affiliations:** 1 Cancer Research UK, Department of Epidemiology, Mathematics and Statistics, Wolfson Institute of Preventive Medicine, Charterhouse Square, London EC1M 6BQ, UK; 2 MRC Epidemiology Unit, Strangeways Research Laboratory, Worts Causeway, Cambridge, UK; 3 Singapore General Hospital, Outram Rd, Singapore 169608, Singapore; 4 National Cancer Centre, Singapore 169610, Singapore

**Keywords:** breast cancer, breast density, Tabar classification, risk factors, interaction

## Abstract

The question of interactions between breast density and other breast cancer risk factors is of interest, since it bears upon the use of density as a marker for changes in breast cancer risk. We studied breast parenchymal patterns and 13 other potential risk factors for breast cancer in 172 breast cancer cases and 338 age-matched controls in Singapore. Dense breast patterns were defined as having Tabar parenchymal pattern IV or V. We found significant interactions between dense patterns and ethnic group (*P*=0.046), and between dense patterns and number of deliveries (*P*=0.04). Among women with nondense breast patterns, the non-Chinese had lower risk than the Chinese with an odds ratio (OR) of 0.47 (95% CI 0.24, 0.88), whereas in those with dense patterns, the non-Chinese had considerably higher risks (OR=5.34, 95% CI 0.54, 52.51). Alternatively expressed, the increased risk with dense patterns was only observed in the non-Chinese (OR=13.99, 95% CI 1.33, 146.99). Among parous women, the protective effect of three or more deliveries was only observed in those with dense breast patterns (OR=0.21, 95% CI 0.06, 0.70). Suggestive but nonsignificant interactions with dense patterns were observed for ever having delivered, age at first delivery, breast feeding and body mass index. The results are consistent with dense breast patterns as a marker for hormonal modification of breast cancer risk.

In recent years, there has been increasing interest in breast density as a risk factor for breast cancer, as a potential marker for changes in the breast and as a criterion for inclusion in prevention trials (Warner *et al*, 1992; Sala *et al*, 1998; Harrison *et al*, 2002; Warwick *et al*, 2003). Radiologically dense breasts are known to be associated with increased risk of breast cancer (Warner *et al*, 1992; Sala *et al*, 1998), to be associated with other risk factors for breast cancer (Jakes *et al*, 2000; Warwick *et al*, 2003) and to be amenable to exogenous hormonal stimuli (Atkinson *et al*, 1999; Atkinson and Bingham, 2002; Greendale *et al*, 2003).

The last phenomenon is of particular interest. Increased density (i.e. high-risk pattern) is associated with preparations such as hormone replacement therapy, which increases exposure to oestrogens (Greendale *et al*, 2003) and therefore risk of breast cancer, and decreased density is associated with preparations such as tamoxifen or isoflavones, which reduce oestrogen exposure and breast cancer risk (Atkinson *et al*, 1999; Greendale *et al*, 2003). This suggests that breast density may be of use as an interim marker of response to hormonal chemoprevention therapy. The fundamental issue to be resolved is whether an induced change in breast density automatically confers the corresponding change in breast cancer risk. This may be ascertained by substudies within the chemoprevention trials such as IBIS (Cuzick *et al*, 2002; Warwick *et al*, 2003). In the meantime, however, some clues can be gained by studying the interrelationships of dense breast patterns, hormonal and other risk factors, and occurrence of breast cancer.

In a previous paper reporting on dense breast patterns and breast cancer risk in a case–control study in Singapore, we noted a significant effect on risk of the dense Tabar IV pattern (Gram *et al*, 1997; Jakes *et al*, 2000) with an odds ratio (OR) estimate of 2.59. We also found that adjustment for traditional risk factors such as parity and age at first birth made little difference to the estimated effect of breast pattern (and conversely adjusting for breast pattern made little difference to the effects of other risk factors). Finally, we found that the Tabar IV pattern was significantly negatively related to parity, positively related to higher educational status, and although not significant, at least suggestively related to other risk factors such as age at first birth, breast feeding history and use of hormone replacement therapy. With both dense patterns and risk factors associated with breast cancer risk and with each other, but with no substantial attenuation of effects when mutually adjusted, this led us to hypothesise that there may be interactions (modification of the effect of traditional risk factors with certain categories of density or *vice versa*). For example, if the protective effect of an early first birth was only manifested in those who showed a corresponding reduction in breast density, this would be further evidence of density as a marker of progression towards breast cancer and would give further support to investigating its potential as an early indictor of the effectiveness of chemoprevention. We therefore propose to investigate interactions between dense breast patterns and other risk factors within the Singapore case–control study.

## MATERIAL AND METHODS

Within the Singapore Breast Screening Project (Ng *et al*, 1998), we conducted a case–control study, with 174 breast cancer cases and 348 controls, matched for age and screening status (Jakes *et al*, 2000). For each case and control, we had data on reproductive history, socioeconomic status as represented by education and occupation, and racial group. We also retrieved the most recent mammogram for each case and control. These were read blind to case–control status for Tabar mammographic pattern by a senior radiologist (FCN). The Tabar pattern has five categories, with patterns IV and V corresponding to the highest density. A full description of the Tabar mammographic pattern is given by Gram *et al* (1997). More details of the Singapore case–control study can be found in Jakes *et al* (2000).

The primary aim of the present study is to evaluate interactions between dense breast patterns and the traditional breast cancer risk factors, for example, is the effect of age at first birth stronger or weaker in women with dense patterns than in women with nondense? In the case–control study, the highest risk category was Tabar IV (Jakes *et al*, 2000). For purposes of interaction analysis, we combined Tabar categories IV and V, because use of Tabar IV alone would lead to very small numbers when crosstabulated simultaneously with other risk factors and case–control status, Tabar pattern V is if anything more dense than IV, and because others have found strong associations of traditional markers of breast cancer risk with both patterns IV and V (Gram *et al*, 1995, 1997).

Statistical analysis was by conditional logistic regression (Breslow and Day, 1980). We tested for interactions between traditional breast cancer risk factors and dense mammographic patterns, and estimated the corresponding OR and 95% confidence intervals (CI) within categories of mammographic patterns.

## RESULTS

Tabar pattern was assessable from the mammograms of 172 (99%) cases and 338 (97%) controls. Table 1

**Table 1:** Tabar mammographic pattern by case–control status

**Tabar pattern**	**Cases (%)**	**Controls (%)**
I	105 (61)	198 (59)
II	15 (9)	67 (20)
III	12 (7)	15 (4)
IV	23 (13)	20 (6)
V	17 (10)	38 (11)
Nondense (I–III)	132 (77)	280 (83)
Dense (IV–V)	40 (23)	58 (17)
Total	172	338

shows Tabar pattern by case–control status. As reported previously, the cases were more likely to have dense patterns than the controls. The numbers of subjects classified by the triple crosstabulation of breast pattern, risk factor and case–control status is given in the appendix.

The results of interaction analyses are shown in Table 2

**Table 2:** Results of analyses of potential interactions between breast patterns and other risk factors

		**ORs (95% CI) for interaction model**			
**Risk factor**	**Category**	**Nondense**	**Dense**	**OR (95% CI) dense only**	***P* for interaction**
Age	<55	1.00 (−)	1.69 (0.70,4.07)	1.00 (−)	0.8
	55+	1.23 (0.33,4.44)	1.77 (0.44,2.05)	1.05 (0.25,4.35)	
Ethnic group	Chinese	1.00 (−)	1.23 (0.74,6.84)	1.00 (−)	0.046
	Other	0.47 (0.24,0.88)	6.57 (0.67,64.07)	5.34 (0.54,52.51)	
Age at menarche	<15	1.00 (−)	1.45 (0.77,2.73)	1.00 (−)	0.8
	15+	0.71 (0.46,1.09)	1.16 (0.57,2.35)	0.80 (0.34,1.88)	
Ever delivered	No	1.00 (−)	0.87 (0.29,2.58)	1.00 (−)	0.3
	Yes	0.34 (0.17,0.69)	0.53 (0.24,1.17)	0.60 (0.22,1.64)	
No of deliveries^a^	1–2	1.00 (−)	4.35 (1.44,13.16)	1.00 (−)	0.04
	3+	0.85 (0.49,1.47)	0.92 (0.41,2.05)	0.21 (0.06,0.70)	
Age at first delivery^a^	<35	1.00 (−)	1.55 (0.85,2.80)	1.00 (−)	0.6
	35+	1.26 (0.29,5.42)	4.02 (0.73,21.95)	2.59 (0.42,15.62)	
Ever breast fed	No	1.00 (−)	1.09 (0.48,2.45)	1.00 (−)	0.3
	Yes	0.39 (0.22,0.68)	0.81 (0.33,1.99)	0.74 (0.27,2.01)	
Menopausal status	Pre-	1.00 (−)	2.79 (0.86,9.02)	1.00 (−)	0.2
	Post-	0.87 (0.38,1.95)	1.14 (0.45,2.92)	0.41 (0.15,1.14)	
Oral contraceptive	Never	1.00 (−)	1.67 (0.93,3.00)	1.00 (−)	0.5
	Ever	0.98 (0.63,1.51)	1.15 (0.50,2.64)	0.69 (0.27,1.77)	
HRT	Never	1.00 (−)	1.51 (0.90,2.53)	1.00 (−)	0.9
	Ever	0.94 (0.50,1.77)	1.43 (0.53,3.78)	0.95 (0.33,2.65)	
Ever married	No	1.00 (−)	0.79 (0.15,4.02)	1.00 (−)	0.5
	Yes	0.30 (0.11,0.84)	0.46 (0.15,1.40)	0.58 (0.14,2.36)	
Formal education	None	1.00 (−)	1.73 (0.89,3.35)	1.00 (−)	0.6
	Any	0.97 (0.63,1.49)	1.29 (0.63,2.62)	0.75 (0.33,1.71)	
Occupation	Housewife	1.00 (−)	1.98 (1.04,3.74)	1.00 (−)	0.2
	Other	1.21 (0.78,1.89)	1.25 (0.59,2.58)	0.63 (0.25,1.55)	
BMI	<25	1.00 (−)	1.31 (0.74,2.30)	1.00 (−)	0.2
	25+	0.98 (0.65,1.48)	2.30 (0.98,5.40)	1.76 (0.71,4.32)	

a Parous women only.

. Results are presented as density-specific effects of each risk factor dichotomised to avoid small numbers in cells, accompanied by formal tests for interaction. The third and fourth columns of the table show the results for the two-way interaction model. The third and fifth columns can be directly compared, since they show the effects of the respective risk factors within the nondense and the dense patterns separately.

The results of the interaction tests indicate that there is significant heterogeneity between dense and nondense patterns, of the effect of number of deliveries on breast cancer risk (*P*=0.04), and of the effect of racial group (*P*=0.046). Among parous women, the reduction in risk with large numbers of children is only seen in those with dense patterns. Among women with dense patterns, a strong increased risk for non-Chinese women was apparent, but for women with nondense patterns, the Chinese women had significantly higher risk than the non-Chinese. This can be expressed in terms of the increased risk with dense patterns: among non-Chinese women there were very few women with dense patterns, but there was a substantial increase in risk associated with these, with an OR of 13.99 (95% CI 1.33, 146.99); no such increase in risk with dense patterns was observed among Chinese women.

No other significant interactions with dense patterns were observed, although potentially interesting nonsignificant interactions were noted for menopausal status, for which an increased risk with dense patterns was only observed for premenopausal women, and body mass index (BMI), for which the increased risk with dense patterns was observed only for those with high BMI (alternatively expressed, the increased risk with high BMI is only seen in those with dense patterns).

## DISCUSSION

The analysis above was prompted by the results of the initial case–control study and should therefore be regarded as hypothesis-generating rather than definitive. It does, however, give rise to some interesting speculations.

In terms of the suggestive heterogeneity of the effect of racial group, this may be a chance effect of the small numbers of women with dense patterns in the non-Chinese racial groups. Whereas substantial numbers of Chinese women had dense patterns (see the appendix), only three cases and one control among the non-Chinese had dense patterns. This difference is highly significant (*P*=0.009). The interesting and reliable feature of this result is that there was no significant increased risk with dense patterns in Chinese women, despite the relatively high prevalence of such patterns in this group (OR=1.23, 95% CI 0.74, 6.84). A possible explanation may be that the high prevalence of dense breast tissue in Chinese women indicates that density in this population reflects body habitus and is not necessarily a feature of a pathological process. Ursin *et al* (2003), however, found an increased risk with density in Asian-American women, who were presumably mostly Chinese- and Japanese-American. In a study in Hawaii, Maskarinec *et al* (2001) found that Chinese and Japanese women had a lower absolute area of mammographic density than Caucasian, Philippine or native Hawaiian women, but due to their smaller breast size, had a higher percentage density (which would correspond more closely to the Tabar pattern IV and V than absolute area of density).

The lack of an increased risk with dense patterns in Chinese women is arguably the indicated area for further research from these results. It should be confirmed or refuted in other studies and using other measures such as percent density. The small number of non-Chinese women with dense patterns is probably due to the fact that more of the non-Chinese women were postmenopausal in this study (94% compared with 88%).

The interaction of dense patterns with number of deliveries among parous women is interesting. A similar result was observed by van Gils *et al* (2000), but with respect to the comparison of parous and nulliparous women. One possible interpretation is that the higher risk associated with low parity only occurs when the correspondingly greater oestrogen exposure in the breast is reflected in an increased density. In our study, the results for ever having delivered suggest that the protective effect of a first child is only seen when accompanied by less dense patterns. This result, however, is suggestive rather than significant (*P*=0.1). The increased risk with a late first delivery being confined to those with dense patterns, and the protective effect of breast feeding being confined to nondense are consistent with this, but these interactions are also nonsignificant.

Another nonsignificant but interesting finding is the absence of an increased risk with dense patterns in postmenopausal women. With one exception (Horwitz *et al*, 1984), this does not seem to be observed in other studies (Byrne *et al*, 1995; Kato *et al*, 1995; Ursin *et al*, 2003). It may be a chance finding or it may relate particularly to the population in our study.

The results suggest that further investigation of the interrelationship of breast density, BMI and breast cancer risk is necessary. Although in our study, there was no significant association of BMI with breast cancer risk, high BMI is an established risk factor in postmenopausal women (Key *et al*, 2003). When we reanalysed using postmenopausal women alone, our observed effect of BMI on risk remained the same. High BMI is also known to be associated with lower density (Sala *et al*, 1999) and from the appendix, our results are consistent with this. It may be that the suggestive but nonsignificant interaction in our study of BMI and dense patterns is real and does go some way to explaining the similarity of adjusted and unadjusted effects, despite the apparent negative confounding. If so, a larger study or a more sensitive measure of density is required to observe the interaction as significant. A percentage density estimate is potentially more sensitive (Brisson *et al*, 2003), both in respect of a possible interaction with BMI and for assessment of the effect of density on risk in general. We aim to have the mammograms in this study re-read for percent density in the near future.

In conclusion, there are interesting interactions and possible interactions of dense breast patterns with other breast cancer risk factors. The interaction with number of deliveries is consistent with density as a marker of hormonal alteration of breast cancer risk, and therefore for its potential as an early indicator of hormonal prevention, as are the suggestive but nonsignificant interactions with BMI, age at first delivery and breast feeding. Research to further confirm and quantify these interactions is indicated, using percentage density and larger study sizes.

## Appendix

Three-way crosstabulations of mammographic pattern, case–control status and other risk factors are shown in Table A1

**Table A1:** Three-way crosstabulations of mammographic pattern, case–control status and other risk factors

		**Controls**		**Cases**	
**Risk factor**	**Category**	**Nondense**	**Dense**	**Nondense**	**Dense**
Age	<55	62	27	28	18
	55+	218	31	104	22
Ethnic group	Chinese	222	57	117	37
	Other	58	1	15	3
Age at menarche	<15	147	29	81	23
	15+	133	29	51	17
Ever delivered	No	17	10	21	9
	Yes	263	48	111	31
No of deliveries^a^	1–2	56	16	28	16
	3+	207	32	83	15
Age at first delivery^a^	<35	256	45	108	27
	35+	7	3	3	4
Ever breast fed	No	75	33	64	25
	Yes	205	25	68	15
Menopausal status	Pre-	22	12	11	13
	Post-	258	46	121	27
Oral contraceptive	Never	173	37	84	29
	Ever	107	21	48	11
HRT	Never	245	47	116	33
	Ever	35	11	16	7
Ever married	No	8	5	11	5
	Yes	272	53	121	35
Formal education	None	174	23	83	19
	Any	106	35	49	21
Occupation	Housewife	189	28	84	23
	Other	91	30	48	17
BMI	<25	136	45	63	27
	25+	144	13	69	13

a Parous women only.

.
